# A Standard Algorithm for Reconstruction of Scalp Defects With Simultaneous Free Flaps in an Interdisciplinary Two-Team Approach

**DOI:** 10.3389/fonc.2019.01130

**Published:** 2019-10-25

**Authors:** Jochen Weitz, Christophe Spaas, Klaus-Dietrich Wolff, Bernhard Meyer, Ehab Shiban, Lucas M. Ritschl

**Affiliations:** ^1^Department of Oral and Maxillofacial Surgery, School of Medicine, Technical University of Munich, Klinikum Rechts der Isar, Munich, Germany; ^2^Department of Neurosurgery, School of Medicine, Technical University of Munich, Klinikum rechts der Isar, Munich, Germany; ^3^Neurosurgery Department, University Hospital of Augsburg, Augsburg, Germany

**Keywords:** CAD/CAM implant, scalp reconstruction, microvascular free flap, temporal anastomosis, squamous cell carcinoma

## Abstract

Reconstructions of complex scalp after ablative resection or by post-traumatic tissue loss, can present difficulties regarding recipient vessel selection, functional, and aesthetic outcome. The harvesting method for many microvascular free flaps requires a need for changing patients position during surgery and makes a simultaneous interdisciplinary two-team approach complicated, which is a major disadvantage regarding safety and operation time. The ideal flap for scalp reconstruction has yet to be described, although the microvascular latissimus dorsi flap is frequently referred to as the first choice in this context, especially after resection of large defects. The purpose of this study is to compare two different microvascular free flaps for a simultaneous scalp reconstruction in an interdisciplinary two-team approach applying a standardized algorithm. All consecutively operated complex scalp defects after ablative surgery from April 2017 until August 2018 were included in this retrospective study. The indications were divided into neoplasm or wound healing disorder. Two microvascular flaps (latissimus dorsi or parascapular flap) were used to cover the soft tissue component of the resulting defects. Seventeen patients met the inclusion criterion and were treated in an interdisciplinary two-team approach. Skull reconstruction with a CAD/CAM implant was performed in 10 cases of which four were in a secondary stage. Nine patients received a parascapular flap and eight patients were treated with latissimus dorsi flap with split thickness skin graft. Anastomosis was performed with no exception to the temporal vessels. One parascapular flap had venous insufficiency after 1 week followed by flap loss. One latissimus dorsi flap had necrosis of the serratus part of the flap. All other flaps healed uneventful and could be further treated with adjuvant therapy or CAD/CAM calvarial implants. Regarding overall complications, flap related complications, flap loss, and inpatient stay no statistical differences were seen between the diagnosis or type of reconstruction. The parascapular flap seems to be a good alternative for reconstruction of complex tumor defects of the scalp besides the latissimus dorsi flap. Stable long-term results and little donor site morbidity are enabled with good aesthetic outcomes and shorter operation time in an interdisciplinary two-team approach.

## Introduction

Scalp defects often arise after ablative tumor surgery of intra- or extracranial neoplasms or in terms of a wound healing disorder secondarily to previous therapy. Small defects can be reconstructed with local flaps as long as a tension free wound closure is possible, which is one of the most critical risk factor for wound healing disorders and secondary revisions (1). Therefore, larger defects (>25 cm^2^) require microvascular free flap transfer for reconstruction with or without computer aided design and computer aided manufactured (CAD/CAM) calvarial implants for accompanying bone defects (2, 3). Craniotomy, to relieve intracranial pressure or to obtain an adequate exposure to certain parts of the cranial vault, is often performed because of brain infarction, intracranial hemorrhages or intracranial disorders caused by tumors and infection (3). Local infection may arise in 1.1–10.0% after reimplantation of the cranial bone flap, which leads to the loss of the bone fragment as well as the covering soft tissue (4, 5). Also tumor invasion of the skull can lead to large cranial bone defects.

The surrounding soft tissues are often inadequate for primary closure apart from reconstruction of the cranial bony contour. In this context vascularized tissue and especially microvascular free flap transfer can overcome this problem. Microsurgical reconstruction is reported to be a save procedure in young and elderly patients (6, 7), but nonetheless the ideal free flap for scalp reconstruction has yet to be described. The common difficulties that accompany and aggravate the soft tissue reconstruction can be subclassified in anatomical, pre-, intra- or postoperative logistics, and patient's and relative's satisfaction. The availability and quality of adequate recipient vessels and surrounding tissue can be altered due to a history of multiple surgical procedures or radiation therapy. For some microvascular free flaps the patient's position must be changed intraoperatively. This maneuver (re-positioning and re-prepping) is time consuming and holds the danger of intubation tube dislocation. Further a simultaneous two-team approach might be hindered.

The purpose of this retrospective analysis is to compare two different microvascular free flaps for a simultaneous scalp reconstruction in an interdisciplinary two-team approach. Further we want to describe our considerations for free flap selection and associated potential pitfalls resulting in a treatment algorithm for clinical practice, as seen for other defect localizations (8, 9).

## Materials and Methods

### Ethical Statement and Enrolled Patients

All clinical investigations and procedures were conducted according to the principles expressed in the Declaration of Helsinki. The study design was reviewed and approved by the ethical committee of the medical faculty of the Technische Universität München. A written informed consent was obtained from all patients.

All patients from April 2017 until August 2018 with a scalp defect that required a microvascular free flap reconstruction in an interdisciplinary approach were included in this retrospective analysis. These were patients with an expected extensive scalp defect or after several unsuccessful attempts of coverage with local flaps. The patients characteristics are summarized in Table 1. The medical records were reviewed for gender, age, initial diagnosis which led to the scalp defect, localization of the defect, usage of a CAD/CAM calvarial implant [titanium or polyetheretherketon (PEEK)], type of microvascular free flap (parascapular or latissimus dorsi), recipient vessels selection, inpatient stay, and incidence of short-term complications. Latter was further subclassified in minor (small dehiscence with no need for surgical revision or conversion to the temporal vessels on the opposite site) or major complications (total or partial flap failure, postoperative hematoma of the reconstructed scalp which required surgical intervention, anaphylactic shock, and death of the patient). Additionally, dehiscence, hematoma, and (total or partial) flap loss were rated as flap related complications.

**Table 1:** Characteristics of analyzed patients.

**No**.	**Gender**	**Age**	**Diagnosis**	**Defect size [cm]**	**Localization of the defect**	**CAD/CAM**	**Scalp reconstruction**	**Microvascular anastomosis**	**Complications**
1	M	73	Meningioma	≤12	Tempero-parietal left	Secondary phase	Parascapular flap	ST A/V left	None
2	M	62	Meningioma	≤12	Parietal left	Secondary phase	Parascapular flap	ST A/V left	None
3	M	75	SCC scalp	>12	Fronto-temporal left	Secondary phase	Latissimus dorsi flap with STSG	ST A/V left	None
4	F	53	SAB	≤12	Temporal left	Titanium	Parascapular flap	ST A/V right	Conversion from left to right temporal vessels Postoperative dehiscence of the flap
5	M	69	Fibroxanthoma scalp	>12	Occipito-parietal left	Secondary phase	Latissimus dorsi flap with STSG	ST A/V left	None
6	M	28	SAB	≤12	Tempero-parietal left	No skull reconstruction	Parascapular flap	ST A/V left	None
7	M	61	SCC scalp	>12	Fronto-temporal right	PEEK	Latissimus dorsi flap with STSG	ST A/V right	None
8	M	88	SCC scalp	>12	Capitulum	(Titanium mesh)	Latissimus dorsi flap and serratus anterior muscle with STSG	ST A/V left	Necrosis of serratus part of latissimus dorsi flap ALT flap for secondary reconstruction
9	F	68	SCC sinus frontalis	≤12	Fronto-temporal right	Titanium	Parascapular flap	ST A/V right	None
10	M	57	Glioblastoma	≤12	Temporal right	Titanium	Parascapular flap	ST A/V right	Necrosis of the flap Latissimus dorsi flap with STSG for secondary reconstruction
11	M	68	SCC scalp	>12	Parieto-occipital left	No skull reconstruction	Latissimus dorsi flap with STSG	ST A/V left	Sepsis during recovery with dead of the patient
12	M	77	Melanoma scalp	≤12	Fronto-temporal right	No skull reconstruction	Parascapular flap	ST A/V right	Perioperative anaphylactic shock
13	M	51	Dermatofibrosarcoma scalp	>12	Occipital left	No skull reconstruction	Latissimus dorsi flap with STSG	ST A/V left	Postoperative hematoma of the scalp
14	F	54	SAB	≤12	Tempero-parietal left	Titanium	Parascapular flap	ST A/V left	Dehiscence of the flap
15	F	78	SAB	≤12	Parietal left	Titanium	Parascapular flap	ST A/V left	Postoperative hematoma of the scalp
16	M	29	SAB	>12	Fronto-temporal	Secondary phase	Latissimus dorsi flap with STSG	ST A/V left	None
17	M	76	SCC scalp	>12	Occipito-parietal median	No skull reconstruction	Latissimus dorsi flap with STSG	ST A/V right	None

*SCC, spinocellular carcinoma; SAB, subarachnoid bleeding; F, Female; M, Male; STSG, split thickness skin graft; ALT, antero-lateral thigh flap; ST A/V, superficial temporal artery/vein*.

### Surgical Procedure and Considerations

Preoperatively, palpation and hand-held doppler measurement were performed in every patient to confirm the availability of the superficial temporal artery and vein (ST A/V). CT-angiography for recipient vessel localization was not needed in any case. The localization of recipient vessel and of the resulting defect determined the positioning side of the patient.

For all included cases the patient was in a right or left lateral decubitus position. A neurosurgeon and maxillofacial surgeon performed the resection of the scalp tumor or the necrotic scalp tissue and preparation of the superficial temporal vessels. A bony defect was immediately reconstructed with a CAD/CAM implant (titanium or PEEK), unless it would have compromised neurological recovery due to increased intracranial pressure. In those cases bony reconstruction was performed in a secondary stage.

As a two team approach, at the same time harvesting of a microvascular parascapular or latissimus dorsi flap was performed by another maxillofacial surgeon in the common techniques as described by others (10, 11). The ST A/V were prepared and a tunnel or an extension incision along the defect was made for the tension free vascular pedicle positioning.

**Figure 1:**
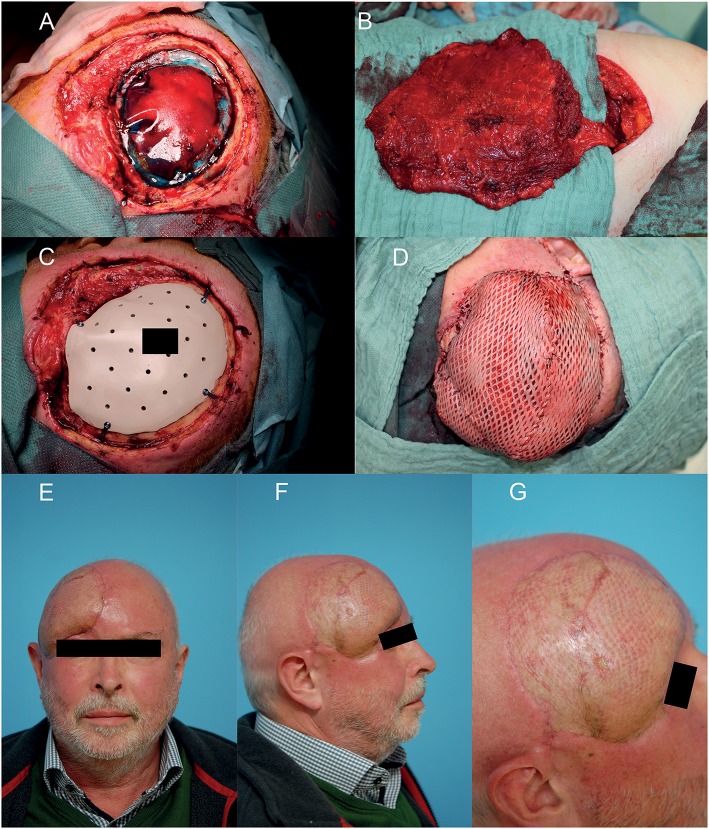
Example of a reconstruction in the fronto-temporal region using a free microvascular latissimus dorsi flap with meshed split thickness skin graft (STSG). **(A)** After interdisciplinary resection of the squamous cell carcinoma and duraplasty of the neurosurgeon. **(B)** Raised free microvascular latissimus dorsi flap in the right decubitus position. **(C)** Bony defect coverage with the patient and defect specific CAD/CAM PEEK-implant. **(D)** Soft tissue coverage with free microvascular latissimus dorsi flap and meshed STSG. **(E–G)** Eighteen months postoperative result. A written informed consent for the publication of the images was obtained from the patient.

Microvascular anastomosis was performed in end-to-end technique, whereby in the case of two comitant veins, one was anastomosed orthograde, the other retrograde to the temporal vein. Then the flap was positioned onto the defect to allow a tension free wound closure. No drainage was put *in situ*. In case of a latissimus dorsi flap, a meshed split thickness skin graft (STSG) was used as skin layer which was sutured onto the muscle flap (12) and additionally fixed with a fibrin sealant spray application (Tisseel, Baxter, Illinois, U.S.) (Figure 1).

### Statistical Analyses

Statistical analysis was carried out by using the “Standard Package for the Social Science” (SPSS for Mac, release 22.0.0, 2013; SPSS Inc., Chicago, IL, USA). Comparisons between reconstruction type (parascapular vs. latissimus dorsi flap) and indication (malignancy vs. wound healing disorder) were performed with the Mann–Whitney-*U*-test. Univariate logistic regression analyses was performed for overall complication rate and inpatient stay. No complementary multivariate logistic regression analysis was performed, because no instance of significance was found in the univariate logistic regression analyses.

All statistical tests were performed at the 0.05 statistical level. *P*-values were two-sided and subjected to a global significance level of 0.05.

## Results

### Descriptive Analysis

Seventeen consecutively treated cases were included in this retrospective study. Male-to-female distribution was 13/4 and the overall median age was 68 years (28–88). The overall median inpatient stay was 10 days (6–44).

The distribution of age, gender, diagnosis, defect localization, applied technique for calvarial bone reconstruction and complications are presented in Table 1.

A comparative descriptive and statistical analysis between parascapular and latissimus dorsi flap is shown in Table 2. Nine patients received a parascapular free flap for scalp reconstruction. Herein, necrosis of the flap occurred in one patient after several attempts to salvage the flap such as a conversion to the facial vein with a vein graft and interim recovery after venous congestion. Secondary reconstruction of the defect was done with a microvascular latissimus dorsi flap with a STSG.

**Table 2:** Comparative descriptive and statistical analysis for both reconstruction types.

**Parameter**	**Parascapular (*n* = 9)**	**Latissimus dorsi (*n* = 8)**	***p*-value**
Age median (range)	62 (28–78)	68.5 (29–88)	0.665
Diagnosis WHD (%)	8 (88.9)	2 (25.0)	0.01^*^
Simul. skull reco. (%)	6 (66.7)	2 (25.0)	0.096
Operation time [min.]	445 (300–673)	432 (401–782)	0.847
Overall complications (%)	5 (55.6)	3 (37.5)	0.47
Flap related complications (%)	4 (44.4)	2 (25.0)	0.693
Total flap loss (%)	1 (11.1)	0 (0.0)	0.346
Inpatient stay [days] median (range)	10 (6–44)	11 (6–30)	0.772

WHD, wound healing disorder; simul., simultaneous; reco., reconstruction.

Mann–Whitney-U-Test;

* *p-value of <0.05 was considered statistically significant*.

Microvascular latissimus dorsi flap with a STSG was used for primary scalp reconstruction in 8 patients in total. There was no total flap failure in this reconstruction group but in case partial flap loss (serratus anterior muscle part) was registered. The resulting defect was reconstructed with an anterolateral thigh (ALT) flap, which healed uneventful.

In all cases the ST A/V were used as recipient vessels. No difficulties were encountered except for one case, in which conversion to the other side was performed because of insufficient flow of the left ST V.

Minor complications (each small dehiscence) were registered in two patients of the parascapular group. It was treated with re-stitching of the flap under local anesthesia and healed uneventful in the follow-up. Major complications were seen in six patients of which one patient died of multi organ failure, one had a perioperative anaphylactic shock, induced by a hydroxyethyl starch (HES) infusion, which was treated uneventful. Two patients had a hematoma which required surgical exploration, one patient had a complete failure of the flap due to venous congestion and another patient had partial failure of the flap.

### Statistical Analysis

The distribution of diagnosis was significantly different in the comparison of the used microvascular flap type (*p* = 0.01, Table 2). Wound healing disorder was the leading indication in the parascapular group (*n* = 8 = 88.9%) and malignancy was the leading indication in the latissimus dorsi group (*n* = 6 = 75.0%). Flap related complications, total flap loss and inpatient stay varied between both reconstructive methods but showed no significant difference for any parameter (*p* = 0.693, *p* = 0.346, and *p* = 0.772), respectively (Table 2).

The distribution of the flap type was significantly different in the comparison of the diagnosis (*p* = 0.01, Table 3), respectively. Overall, six out of seven malignancies were reconstructed with the latissimus dorsi flap. Vice-versa eight out of 10 wound healing disorders were reconstructed with the parascapular flap. Bone defects of patients with a wound healing disorder were more often primarily reconstructed (60%) than patients with a malignancy (28.6%; *p* = 0.215, Table 3). Flap related complications, total flap loss and inpatient stay varied between both underlaying diagnoses but showed no significant difference for any parameter (*p* = 0. 127, *p* = 0. 403, and *p* = 0. 845), respectively (Table 2).

**Table 3:** Comparative descriptive and statistical analysis for both indications (malignancy vs. wound healing disorder).

**Parameter**	**Malignancy (*n* = 7)**	**WHD (*n* = 10)**	***p*-value**
Age median (range)	75 (51–88)	59.5 (28–28)	0.13
Flap type parascapular (%)	1 (14.3)	8 (80.0)	0.01^*^
Simul. skull reco. (%)	2 (28.6)	6 (60.0)	0.215
Operation time [min.]	430 (401–782)	440 (300–673)	0.626
Overall complications (%)	4 (57.1)	4 (40.0)	0.499
Flap related complications (%)	2 (28.6)	4 (40.0)	0.127
Total flap loss (%)	0 (0.0)	1 (10.0)	0.403
Inpatient stay [days] median (range)	10 (6–30)	11.0 (6–44)	0.845

WHD, wound healing disorder; simul., simultaneous; reco., reconstruction.

Mann–Whitney-U-Test;

* *p-value of <0.05 was considered statistically significant*.

Univariate logistic regression analysis showed no significance for any parameter on the overall complication rate and inpatient stay (Table 4).

**Table 4:** Univariate logistic regression analyses for the overall incidence of complications and inpatient stay.

	**Overall complications**		**Inpatient stay**	
**Parameter**	***p*-value**	**95%-CI**	***p*-value**	**95%-CI**
Age	0.496	−0.011–0.023	0.479	−0.221–0.449
Gender	0.225	−0.981–0.25	0.985	−12.882–12.651
Diagnosis	0.517	−0.379–0.722	0.894	−11.697–10.297
Flap type	0.488	−0.722–0.361	0.881	−11.619–10.063
Operation time	0.841	−0.002–0.003	0.328	−0.066–0.023
Simultaneous skull reconstruction	0.256	−0.235–0.818	0.097	−1.674–18.063
Overall complications	/	/	0.077	−1.078–18.142

*95%-CI, 95% confidence interval*.

## Discussion

For reconstruction of scalp defects of 25 cm^2^ or more, especially if the defect is located close to the hairline or alloplastic materials need to be covered, free tissue transfer is required (12, 13). In the past decades several free flaps were described to reconstruct the scalp. In this context, defect size, recipient vessel, and pedicle length are the main factors, that contribute to the choice of flap type. The latissimus dorsi flap with a STSG is frequently referred to as the first choice in reconstruction of large scalp defects (2, 6, 12, 14). The ALT flap can be used as an alternative, but this microvascular flap is associated with anatomical variations, bulkiness if it is raised as a non-perforator flap and the patient needs to be re-positioned intraoperatively in many cases, which prevents a two team approach (6, 15). The pedicle length is described to be excellent and also allows microvascular anastomoses to the facial artery and vein (16). Uzun et al. compared musculocutaneous (latissimus dorsi and rectus abdominis) and fasciocutaneous (ALT and radial forearm) flaps for the coverage of composite scalp defects (17). They reported a less atrophy and less blood loss in the fasciocutaneous flap group. For these reasons, we chose the ALT flap for secondary reconstruction, when the latissimus dorsi flap failed partially in one patient. Alternatively, the ALT flap can also be used as a first choice flap in defects with a ≤12 cm diameter.

**Figure 2:**
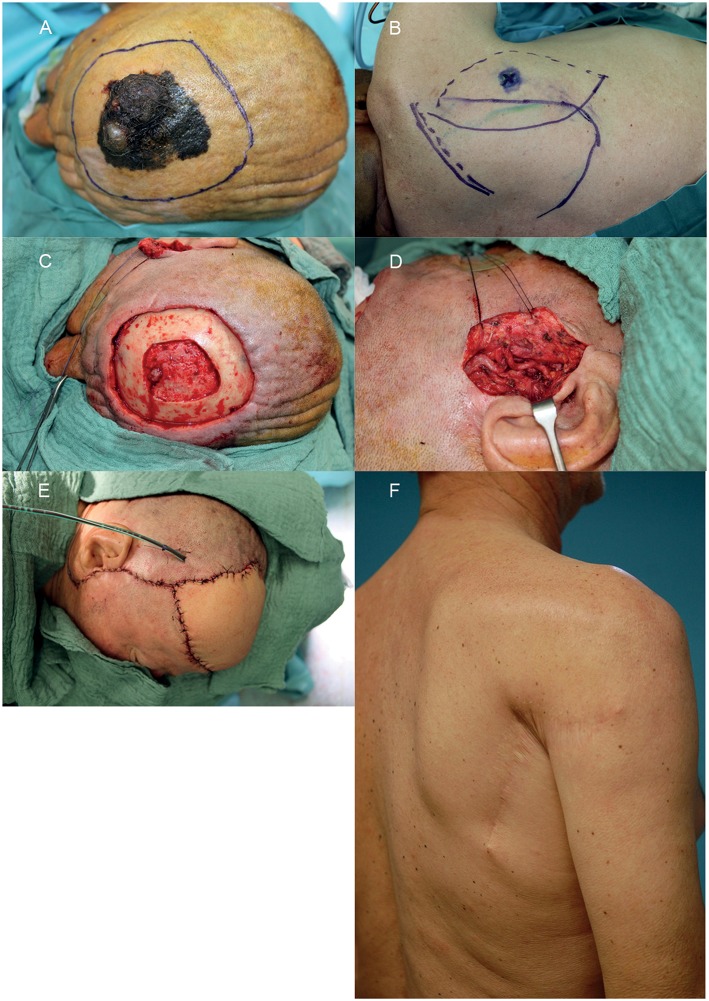
Example of a reconstruction in the fronto-parietal region using a parascapular flap. **(A)** Intended resection margins of melanoma. **(B)** Donor site with marked triangular space. **(C)** Soft and hard tissue defect after interdisciplinary resection. **(D)** Prepared temporal vessels. **(E)** Defect reconstructed with parascapular flap from the ipsilateral side. **(F)** Donor site on the right back 1 year postoperative. A written informed consent for the publication of the images was obtained from the patient.

**Figure 3:**
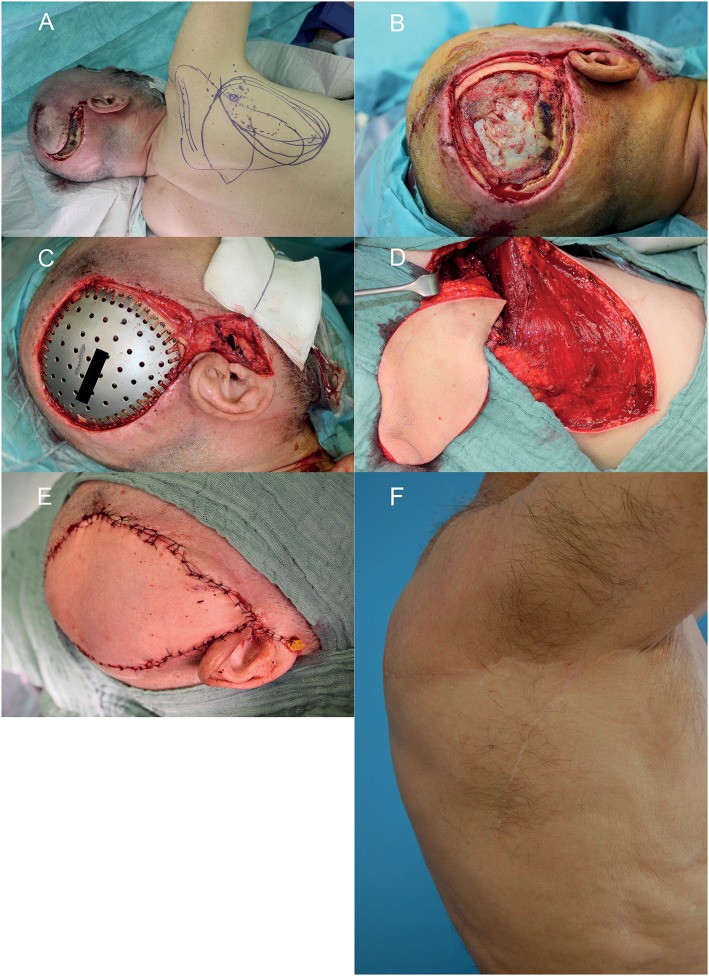
Wound healing disorder after resection of a glioblastoma multiforme relapse in the right temporal region. **(A)** Wound situation and planning of the microvascular parascapular flap in the left decubitus position. **(B)** After debridement of the wound. **(C)** After insertion of the CAD/CAM titanium implant. **(D)** The microvascular parascapular flap with pedicle in the donor site. **(E)** Immediate reconstructive result after soft tissue closure. **(F)** donor site on the right back 1 year postoperative. A written informed consent for the publication of the images was obtained from the patient.

**Figure 4:**
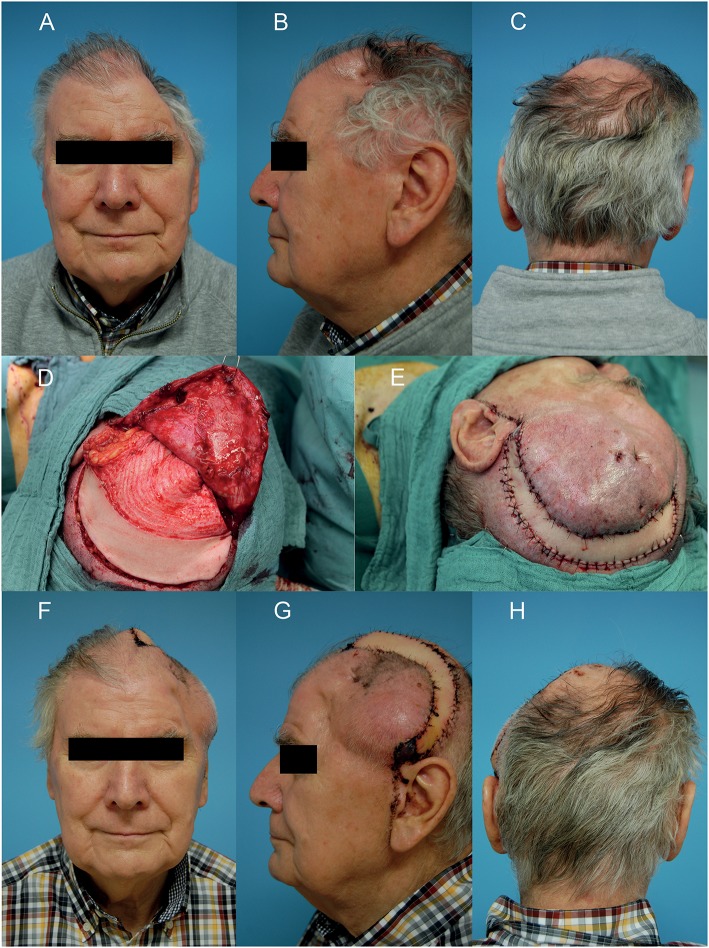
Wound healing disorder after two times resection of a meningioma in the left temporo-parietal region. **(A–C)** Preoperative situation of the defect in frontal, side, and back view. **(D)** After microvascular anastomosis and de-epithelialization of the anterior part of the parascapular flap for soft tissue release and to reduce temporal hollowing. **(E)** After wound closure. **(F–H)** Clinical situation on the 7th postoperative day in frontal, side, and back view. A written informed consent for the publication of the images was obtained from the patient.

**Figure 5:**
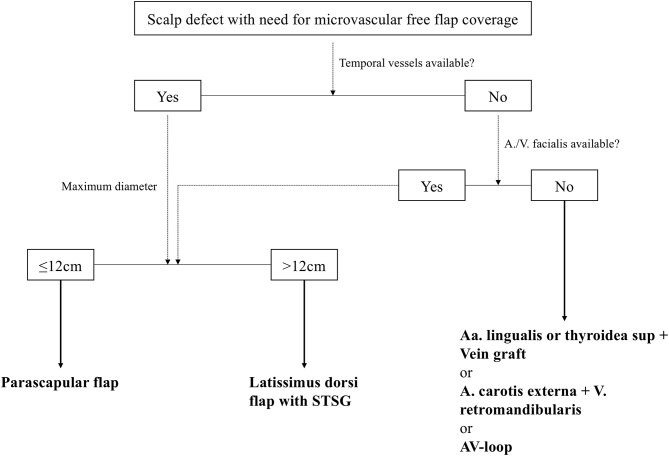
Standardized reconstructive algorithm for scalp defects, that need a defect coverage with a microvascular free flap. STSG, split-thickness skin graft; AV-loop, arteriovenous loop.

According to our interdisciplinary experience we propose an algorithm for scalp reconstruction where the parascapular flap is the standard flap for reconstruction after wound healing disorders, small neoplasms (diameter ≤12 cm and along oval soft tissue defect), loss of calvarial bone and preparation for calvarial implants (**Figure 5**). We prefer the parascapular flap over the latissimus dorsi free flap due to the reason of maintaining the upper extremity function, which has a significant influence on quality of life, as well as no scaring or muscle atrophy which could jeopardize the scalp and the CAD/CAM-assisted bone reconstruction (18–20) (Figures 2, 3). Klinkenberg et al. described a good patient's satisfaction with the parascapular flap in comparison to the ALT or lateral arm flap (21). Fisher et al. compared patient's satisfaction who received both, ALT and parascapular flap. Herein parascapular flap was also the preferred flap, even though the scar dimensions were greater than with the ALT flap (22). Furthermore, partial flap de-epithelialization can be done (Figure 4). The de-epithelized part can be used to treat temporal hollowing, which is often seen as a postsurgical defect due to temporalis muscle disinsertion/atrophy or superficial temporal fat pad atrophy after coronal incision (23). In this context, the usage of muscular latissimus dorsi flap would not need a de-epithelialization with the same effect on avoiding temporal hollowing.

In very large and predominantly round scalp defects a latissimus dorsi flap with STSG is the primary option for reconstruction in our algorithm as described by others (14). The reason therefore is its potentially large surface area, if the transplant is taken as a muscle flap (19). This cannot be achieved by a parascapular flap with a primary closure of the donor site. If even the latissimus flap is insufficient for more extensive defects, Goertz et al. described the combination of LD and PS as a good and reliable option for these cases (24).

In case of flap failure an ALT flap is preferred as secondary reconstruction method due to low donor site morbidity and its favorable pedicle length, which makes the need for an interposition vein graft unnecessary. Disadvantage is the harvesting in supine position of the patient as well as often being bulky. Thinning of the ALT can be done, but might come along with certain risk for flap failure due to vascular compromise because of vasospasm or injury of the perforator, especially in large ALT flaps (25, 26).

In our opinion the parascapular does not oppose any problems due to be bulky or color mismatch, as reported by van Driel et al. (6).

We had one flap loss and the overall flap survival was 94%, which is in line with the reported data in the literature (6, 14). Although in our study it is mainly an elderly population, no adverse effects due to age were seen, as reported by many authors (13, 20).

The ST A/V, if palpable preoperatively (all cases in our study), were the preferred recipient vessel for anastomosis. It is a reliable vascular system because of its consistent anatomy, proximity to the defect, and sufficient vessel caliber for all microvascular flaps (12, 27). Although the caliber of the superficial vessels can be small, especially the distal part, further dissection proximal into the cranial pole of the parotid gland in front of the tragus can be performed to obtain a bigger caliber for vascular anastomosis (6). The temporal vessels are superior to the facial vessels for anastomosis due to the fact that in case of facial recipient vessels and according to the chosen microvascular flap often a interposition vein graft is required, which is known to be a risk factor for flap survival (28, 29). Further, we are able to perform both microvascular anastomoses of the comitant veins to the ST V. Herein we anastomose the better draining comitant vein orthograde to ST V to achieve a drainage to the deep venous system. The weaker comitant vein is anastomosed to the other end of the ST V to achieve a retrograde drainage to the superficial system. In the rare case that the temporal vessels are not suitable for microvascular anastomosis, the neck vessels are a good backup option, especially facial or thyroid artery and vein. In addition, you have the opportunity to raise a vein graft from the external jugular vein via this approach or to include a AV-loop in a single or two-staged regimen, if this should be necessary (Figure 5) (30, 31).

## Limitations

According to the nature of a retrospective study, there is a potential for variability in reports of clinical data provided by treating clinicians. The authors attempted to minimize the bias. Secondly, patients were recruited from an inpatient setting only between April 2017 until August 2018 in a single university hospital. The enrolled and analyzed cohort was small. Therefore, the patients might not be representative for the entire population requiring a scalp reconstruction. This rather small patient number guarantees on the other a treatment according to the presented algorithm, that might differ, if we had enrolled more patients from the past years. The statistical results should be interpreted more as a trend. But in summary the cohort meets very well the commonly described underlaying diagnosis and associated comorbidities and history of treatment. Third, records did not comprise radiological or photographic findings to sufficiently describe postoperative morphological and aesthetic changes. We plan to implement this in our pre- and postoperative follow-up for the future, including 3D-photography and a health related questionnaire for quality of life.

## Conclusions

The parascapular flap seems to be a good alternative for microvascular reconstruction of complex composite defects of the scalp ≤12 cm with comparable operation time. Stable results and little donor site morbidity are enabled with subjective satisfying aesthetic outcomes an interdisciplinary two-team approach. A practical treatment algorithm is described.

## Data Availability Statement

All datasets generated for this study are included in the manuscript/supplementary files.

## Author Contributions

JW and LR: study conception, major contributor in writing the manuscript, and operations. CS: data analysis and contributor in writing the manuscript. K-DW and BM: operations and study conception. ES: data acquisition and interpretation, statistical analyses, and writing results section. All authors contributed to manuscript revision, read and approved the submitted version.

### Conflict of Interest

The authors declare that the research was conducted in the absence of any commercial or financial relationships that could be construed as a potential conflict of interest.
